# Ventilating Sewers

**Published:** 1875-06

**Authors:** 


VENTILATING SEWERS.—In well ventilated sewers, analysis shows the presence of carbonic acid, nitrogen, sulphuretted hydrogen, and traces of various other gases; but the sewers of New York are not well ventilated. Indeed they are much of the time with no ventilation at all, consequently we may expect to find in them a rare combination of foul gases. Probably the only thing that saves us from a continuous epidemic of typhoid fever is the fact that our sewage is more diluted with water, in proportion to its volume, than that of any other large city. In the use of water we are extravagant to a degree unknown in any part of Europe. Owing to this dilution of our sewage, it is probable that less gas is generated in proportion to the volume of flow than in cities where more attention has been given to this subject; but it is none the less desirable to keep the air of our unventilated sewers from mingling with that of our dwellings. Our sewers are absolutely without ventilation, and the only escape for gases, which are often held under considerable pressure, is through soil and waste pipes. The mouths of the sewers are, as a rule, so placed as to be completely submerged at high tide, at which times the river water forces its way up into them for a considerable distance, compressing the air confined within, in proportion to the resistance offered at the various outlets by which it makes its escape. Under these conditions it is readily seen that when the mouth and man-hole ventilators of a sewer are closed, the confined air is liable to force itself into our dwellings. If there is a pipe rising to the roof and opening there, the pressure is relieved, and there is less danger of sewer-gas finding its way into the house, providing the various parts of the apparatus connected with the sewers, soil-pipes, &c., are arranged properly in other respects.

It is clear that ventilation upwards might be dispensed with, if the sewers are made large, as they are in Paris, and if constant care is taken to keep their flow free and unimpeded throughout the entire length of the sewer-channel. So long as the fall is slight, so long as there is not "head" enough to carry off heavy debris, there will be obstruction of the canal, and this obstruction will permit an accumulation of offensive materials. If we ventilate, it must be by high chimnies, so that the dangerous gases may not be emitted for our use. But most of the sewer-gases are heavy, and therefore the chimney system would decline to take advantage of such an external opening.

Large cities depend greatly upon an abundant water-supply, for immunity from fatal epidemics. Our water should be free as air and sunlight, and no system of measurement which could deprive poor people of a copious supply should for a moment be encouraged. If we can furnish water to an unlimited extent, having all water-pipes connect with sewers of enormous conducting power, we accomplish ventilation most surely. The water not only dilutes the sewage, but it also absorbs the bad gases. Running water compels air-currents in the direction of the stream and secures continuous and ample ventilation.

The sewerage of the great city of

New York, in all but some of the older localities around and below Canal Street, will do very well, so long as the water-supply is unlimited, and not subject to any taxation which shall fall upon the poor, directly. If we would have increasing intelligence and growth in all good qualities, we must have those excellent sanitary conditions which accompany an abundant water-supply. We must not permit the inhabitant of a tenement house to say that uncleanness is permitted because it is cheap. We are wicked if we offer a premium for dirty faces, dirty bodies, and foul linen. We can signalize our careers in no way more worthily than by advocating free water for bathing and for all domestic uses.

